# Spontaneous fistulization of a giant pancreatic pseudocyst into the duodenum following acute pancreatitis: a case report

**DOI:** 10.1097/MS9.0000000000005650

**Published:** 2026-09-08

**Authors:** Bikash Kumar Shah, Adesh Kantha, Abhisekh Bhattarai

**Affiliations:** a Department of General Surgery, Maharajgunj Medical Campus, Institute of Medicine, Tribhuvan University, Kathmandu, Nepal; b Department of General Surgery, Tribhuvan University Teaching Hospital, Kathmandu, Nepal

**Keywords:** acute pancreatitis, cystoduodenal fistula, pancreatic pseudocyst, spontaneous resolution

## Abstract

**Introduction:**

Pancreatic pseudocysts are well-recognized sequelae of pancreatitis, but giant pseudocysts (>10 cm) are uncommon and rarely resolve through spontaneous fistulization. Spontaneous internal rupture into the gastrointestinal tract happens in less than 3% of cases and typically requires surgical or endoscopic intervention.

**Case presentation:**

We report the case of a 40-year-old male with a history of alcohol use who developed a giant pancreatic pseudocyst (22 × 12 cm) following acute pancreatitis. He presented with abdominal discomfort, early satiety, and vomiting. Imaging revealed a massive cyst compressing the stomach and duodenum. Although initially managed conservatively, the patient later experienced episodes of severe abdominal pain and coffee-ground vomiting.

**Clinical discussion:**

Spontaneous internal drainage of a giant pancreatic pseudocyst into the duodenum is very rare. This case highlights the potential for non-operative management in carefully selected, clinically stable patients, despite the size of the lesion and the risk of complications.

**Conclusion:**

We describe an unusual case of a giant pancreatic pseudocyst that resolved spontaneously through cystoduodenal fistulization. Conservative management led to full recovery, underscoring the importance of individualized treatment strategies in pancreatic pseudocyst management.

## Introduction

A pancreatic pseudocyst is defined as a fluid collection that is more than 3 weeks old and is surrounded by a fibrous wall, according to the Atlanta Symposium^[^1^]^. It typically develops following pancreatic ductal disruption due to acute or chronic pancreatitis or pancreatic trauma. Although the overall incidence in adults is low (0.5–1 per 100 000 per year), it occurs more frequently in patients with acute or chronic pancreatitis, and its detection has increased because of newer imaging techniques^[^2^]^. While the risk of malignancy is greater in symptomatic cysts, up to 47% of incidentally detected pancreatic cystic lesions may be malignant or pre-malignant; pancreatic pseudocysts account for 15-30% of these lesions and can present as single or multiple lesions with variable clinical features. Based on the largest diameter, pseudocysts are classified as small (≤2 cm), medium (2–6 cm), large (6–10 cm), and giant (>10 cm)^[^3^]^.

Ultrasonography (USG), computed tomography (CT), and magnetic resonance imaging (MRI) are the imaging techniques employed to diagnose the condition. Transabdominal ultrasonography is typically performed as the initial imaging modality, and a high-sensitivity, high-resolution endoscopic ultrasound (EUS) may be used for further characterization, with the potential to detect cystic lesions smaller than 2 cm^[^4^]^.

HIGHLIGHTS

- Giant pancreatic pseudocysts (>10 cm) are rare complications of pancreatitis in the modern era of imaging and management.
- Spontaneous fistulization of a pseudocyst into the duodenum is extremely uncommon, occurring in fewer than 3% of pseudocysts.
- Our case describes a pseudocyst (22 × 12 cm) following acute pancreatitis that fistulized into the duodenum without surgical intervention.
- Conservative management was successful, with complete resolution demonstrated on follow-up imaging.
- This case highlights the importance of individualized management, as not all giant pseudocysts require invasive intervention if the patient remains clinically stable.

Depending on where it drains, a pancreatic pseudocyst fistula must be managed accordingly. If it drains into the duodenum or colon, surgery might be necessary^[^5^]^. Contemporary guidelines favor a stepwise, increasingly minimally invasive approach: endoscopic ultrasound (EUS)-guided transmural drainage is now generally preferred over surgical drainage as first-line therapy for symptomatic, infected, or enlarging pseudocysts in centers with the requisite expertise, and intervention is typically deferred for approximately 4–6 weeks after the inciting episode of pancreatitis to allow the cyst wall to mature, provided the patient remains clinically stable^[^6^]^. Indications for intervention include persistent symptoms, infection, gastric outlet or biliary obstruction, and hemorrhage; asymptomatic, stable pseudocysts, including large ones, may instead be managed expectantly with serial clinical and radiological monitoring, as illustrated in the present case. Surgical intervention and endoscopic clipping of bleeding vessels are both options for controlling active bleeding. An alternative intervention in cases of bleeding is endoscopic transcatheter arterial embolization, guided by radiological imaging techniques^[^7^]^.

This report aims to highlight the rare occurrence of spontaneous cystoduodenal fistulization of a giant pancreatic pseudocyst and to illustrate the role of conservative, non-operative management in carefully selected, hemodynamically stable patients in contrast to standard guideline-based indications for invasive drainage.

As per the TITAN Guidelines 2025 on the declaration and responsible use of artificial intelligence in academic publishing, we acknowledge that no AI-assisted tools were used in the process of writing the article^[^8^]^.

## Case presentation

A 40-year-old male with a history of alcohol consumption was admitted to our hospital with a 1-month-long history of abdominal pain. During this first admission, he was diagnosed with acute pancreatitis and managed conservatively. His hospital course was uneventful, and he was discharged in stable condition with advice for alcohol cessation, gradual dietary advancement, and follow-up after 6 weeks.

Following discharge, he abstained from alcohol and resumed a normal diet. However, 4 weeks later, he experienced progressively worsening upper abdominal discomfort, followed by epigastric fullness, early satiety, nausea, and intermittent vomiting, and immediately presented to the hospital again.

On this second admission, the patient was hemodynamically stable. Abdominal examination revealed a cystic swelling over the epigastric region measuring approximately 7 cm × 5 cm, with mild tenderness. The rest of the physical and systemic examination was unremarkable. Laboratory evaluation revealed a low ionized calcium level (2.01 mg/dL; reference 4.4–5.2 mg/dL), markedly elevated amylase (339 U/L; reference 40–140 U/L) and lipase (415 U/L; reference 10–140 U/L), hyperbilirubinemia (total bilirubin: 2.5 mg/dL, reference 0.1–1.2 mg/dL; direct bilirubin: 1.0 mg/dL, reference <0.3 mg/dL), elevated AST/SGOT (60 U/L; reference 5–40 U/L), raised alkaline phosphatase (194 IU/L; reference 44–147 IU/L), elevated LDH (350 U/L; reference 140–280 U/L), and leukocytosis (total leukocyte count 12 300/µL; reference 4000–11 000/µL), with hemoglobin remaining within normal limits at 13.3 g/dL.

Trans-abdominal ultrasonography revealed a large cystic lesion in the pancreas compressing nearby structures, and computed tomography (CT) of the abdomen (Fig. 1) demonstrated a giant pancreatic pseudocyst measuring 22 cm × 12 cm arising from the pancreatic body, compressing the stomach superiorly and the duodenum laterally. As the patient remained clinically stable, with no evidence of infection, hemorrhage, or peritonitis, a conservative management approach was adopted.

**Figure 1.**
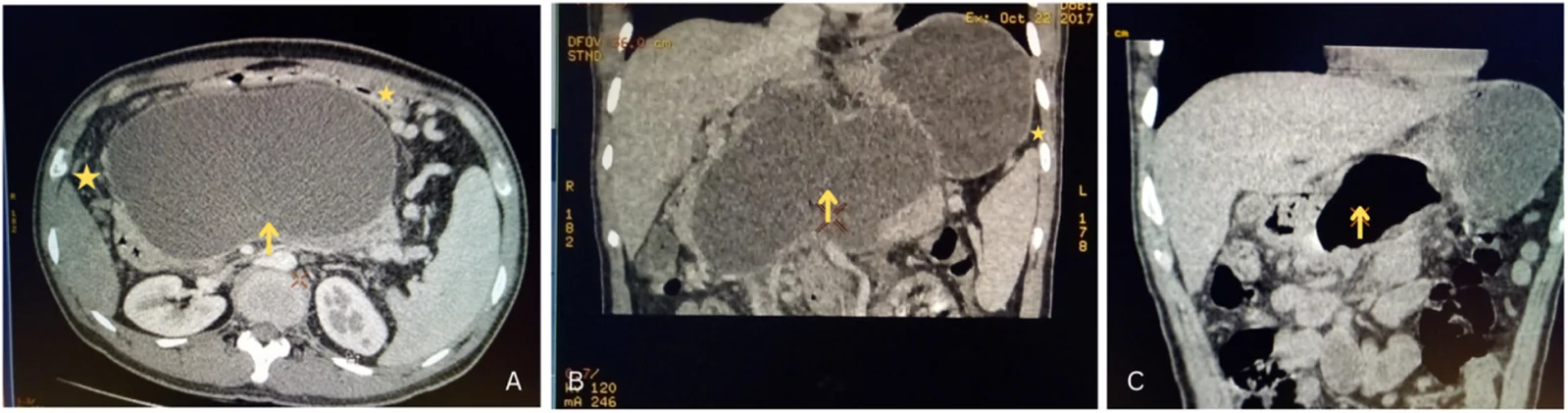
Computed tomography of the abdomen showing a pseudocyst of the pancreas. (A) Giant pseudocyst (arrow) compressing the stomach upward (small asterisk) and the duodenum laterally (large asterisk); (B) large pseudocyst (arrow) causing extrinsic compression of the stomach (asterisk); (C) ruptured pseudocyst (arrow) of the pancreas into the duodenum.

After 5 days of hospitalization, he had multiple episodes of severe abdominal pain followed by dark, coffee-colored vomiting. Given the acuity of this presentation, the differential considerations included active upper gastrointestinal hemorrhage, cyst rupture, and secondary infection. However, the patient remained hemodynamically stable throughout this episode (blood pressure and heart rate within normal limits), with serial hemoglobin measurements declining from 13.3 to 12.8 g/dL and no other clinical or laboratory evidence of active bleeding, which supported continued observation rather than urgent intervention. Repeat CT showed a decreased size of the pseudocyst and suggested a spontaneous cystoduodenal fistula (Fig. 1C). Upper gastrointestinal (UGI) endoscopy showed extrinsic compression of the duodenum by the pseudocyst, with visible leakage of pancreatic juice into the duodenal lumen (Fig. 2), without a visible vessel or evidence of active bleeding on endoscopic examination. Given that the patient remained hemodynamically stable without signs of sepsis, active bleeding, or peritonitis, conservative management was continued, consistent with a strategy of close observation reserved for clinically stable patients even when imaging findings might otherwise prompt intervention. Over the subsequent weeks, his symptoms gradually resolved. A follow-up CT scan performed 6 weeks later demonstrated complete resolution of the pancreatic pseudocyst, and no surgical or endoscopic intervention was required.

**Figure 2.**
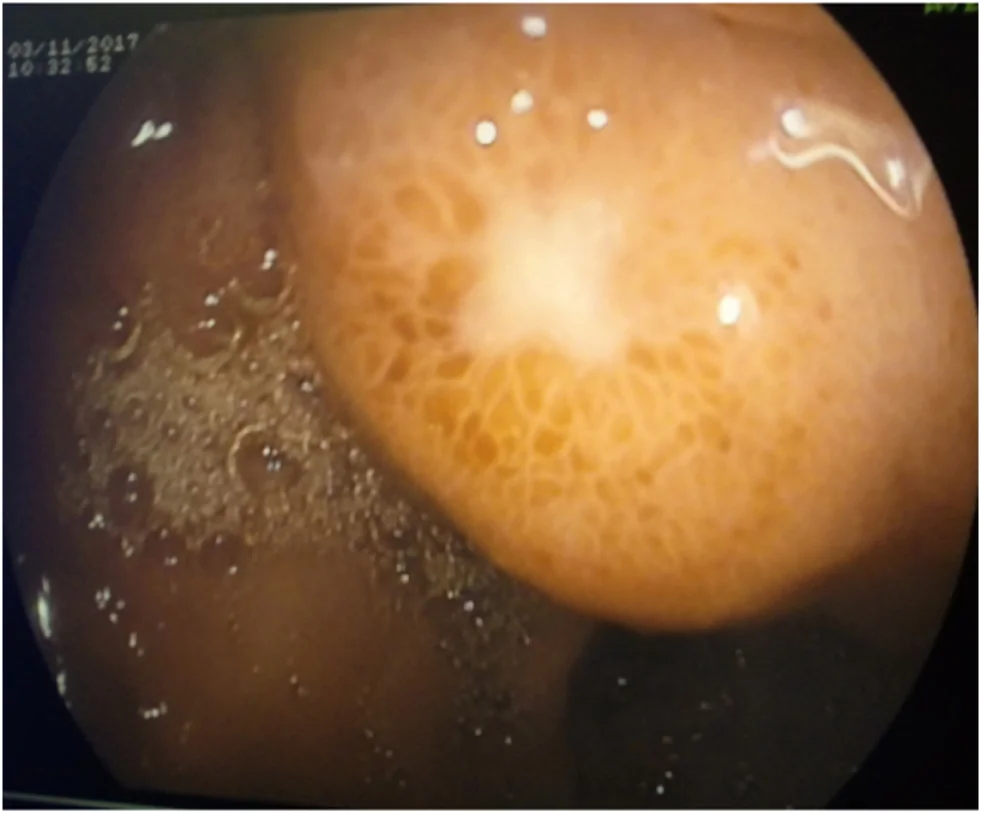
Upper GI endoscopy showing pseudocyst of the pancreas producing compression in duodenum upward with the leakage of pancreatic juice in the duodenal lumen.

The patient had an unremarkable past medical and family history and recovered fully with close monitoring and supportive care.

## Discussion

Pancreatic pseudocysts are histopathologically distinct from true cysts, as they have a fibrous-granulomatous wall as opposed to an epithelial lining. They are further subdivided into four types by the Atlanta classification system: acute fluid collection, acute pseudocysts, chronic pseudocysts, and pancreatic abscess^[^9^]^.

The differential diagnosis of a large pancreatic cystic collection in this clinical setting includes walled-off necrosis (WON), which typically contains heterogeneous necrotic debris and develops later in the disease course (generally more than 4 weeks) following severe or necrotizing pancreatitis, and primary pancreatic cystic neoplasms such as mucinous cystic neoplasm and intraductal papillary mucinous neoplasm, which characteristically demonstrate septations, mural nodules, calcifications, or communication with the pancreatic duct on imaging. In our patient, the homogeneous, well-defined, non-septated appearance of the collection, without mural nodularity or calcification, together with the clear temporal relationship to a preceding episode of acute pancreatitis, favored the diagnosis of a pseudocyst over these alternatives.

Pancreatic pseudocysts are a well-known complication of chronic pancreatitis, occurring in about 25% of patients^[^10^]^. Pseudocysts following acute pancreatitis are relatively uncommon and occur in 5–16% of cases^[^11^]^. A giant pseudocyst is defined as one with its longest diameter greater than 10 cm, and is relatively infrequent these days because of present-day treatment options^[^12^]^.

In acute pancreatitis, there is inflammatory digestion and subsequent liquefaction of the pancreatic parenchyma, which, when encapsulated by granulation tissue, forms a pseudocyst. In chronic pancreatitis, a pseudocyst can be triggered by an acute pancreatitis exacerbation or by ductal disruption. Additionally, pancreatic trauma or injury, ductal damage from surgery, biliary stones, or alcohol use can result in the formation of a pseudocyst.

Most pancreatic pseudocysts are asymptomatic but can occasionally present with abdominal pain, weight loss, or early satiety due to gastric outlet or intestinal obstruction, or with jaundice resulting from biliary obstruction attributable to mass effect. Fistulization is known to occur in less than 3% of pseudocysts, and the biliary tract, stomach, duodenum, renal collecting system, colon, and the bronchial tree may all be affected by spontaneous rupture of a pancreatic pseudocyst^[^13,14^]^. Only 5% of pancreatic pseudocysts are complicated by hemorrhage; however, this condition carries a 40% mortality rate^[^15^]^.

Diagnosis is established based on imaging showing an intrapancreatic or peri-pancreatic fluid collection against a background of chronic pancreatitis or in a patient who has recovered from acute pancreatitis. Although CT scans and MRIs are the primary modalities for evaluating pancreatic cysts, histopathology may be required to rule out malignant cystic lesions, most commonly mucinous cystadenoma^[^6^]^. Since the cyst is clearly defined, homogeneous, and devoid of calcifications and mural nodules, pancreatic cystic neoplasms can be ruled out as the cause based on the results of the CT imaging (Fig. 1A and B)^[^4^]^. Along with our patient’s imaging reports, characteristics like homogeneity, the presence of necrotic fat and a mixture of necrotic cells, and a clearly defined wall all point to the diagnosis of a pancreatic pseudocyst.

Intervention for pancreatic pseudocysts may be required in patients who experience peritonitis, infection, bleeding, or gastric outlet obstruction. Acutely worsening abdominal pain, ongoing nausea or vomiting, insufficient oral intake, fevers, chills, or hypotension are concerning signs that should alert doctors to the possibility of these complications^[^6^]^. Percutaneous, endoscopic, and surgical drainage or excision represent the three types of invasive intervention. To allow the pseudocyst wall time to thicken and mature, any intervention should ideally be postponed until about 6 weeks after the pancreatitis episode that triggered the pseudocyst, assuming there are no complicating factors^[^6^]^. Video-assisted pancreatic necrosectomy and laparoscopic cystogastrostomy have both been used successfully. Cysto-jejunostomy was planned for our patient; however, the cyst spontaneously ruptured into the duodenum, and the patient recovered without complications or need for surgical treatment. Among invasive options, EUS-guided transmural drainage has emerged as the preferred first-line approach over surgical or percutaneous drainage in centers with the requisite expertise, owing to its lower morbidity and comparable efficacy^[^6,16^]^. Our patient met several size and imaging criteria that might typically prompt earlier intervention under such guidelines; however, the absence of infection, hemorrhage, or peritonitis, together with close clinical monitoring, permitted continued observation, which this case suggests can be justified even in giant pseudocysts, provided strict stability criteria are maintained.

It is extremely uncommon to encounter a pancreatic pseudocyst of this size, as was the case in this patient. If left untreated, such pseudocysts can present with complications including infection, hemorrhage, and rupture^[^17^]^. Recent literature, however, suggests a shift toward less invasive management strategies, with endoscopic approaches such as cystodigestive bypass and transpapillary drainage emerging as safe and effective alternatives in selected cases of spontaneous pseudocyst fistulization^[^16^]^. Aksim *et al* similarly reported a case of spontaneous internal fistulization of a pancreatic pseudocyst into the duodenum that, comparable to our patient, resolved without the need for surgical or endoscopic intervention^[^16^]^. Similarly, another case report describes spontaneous pseudocyst rupture occurring after acute pancreatitis that presented with gastric outlet obstruction and was managed conservatively^[^18^]^. Compared with these previously reported cases, which generally involved smaller or moderately sized pseudocysts, the present case is notable for the giant size of the pseudocyst (22 × 12 cm) at the time of spontaneous fistulization; nonetheless, the clinical course and outcome mirrored these reports, with hemodynamic stability maintained throughout and complete resolution achieved without surgical or endoscopic intervention. This suggests that pseudocyst size alone should not preclude a trial of conservative management, provided the patient remains clinically stable and is monitored closely. It may be uncommon for a pancreaticoduodenal fistula to be large enough to be visualized on CT imaging, but in this patient, an air bubble within the collapsing pseudocyst on the repeat CT scan supported the diagnosis of a pancreaticoduodenal fistula. Moreover, this patient illustrates an unusual route to pancreatic pseudocyst resolution via internal fistulization into the duodenum, which sealed spontaneously without requiring surgical intervention, making this a notable and instructive case. The patient was initially anxious about the possible need for surgery, but with clear explanations and close monitoring, he was reassured and went on to recover fully without an operation.

## Conclusion

This case highlights an exceptionally rare presentation of a giant pancreatic pseudocyst that underwent spontaneous fistulization into the duodenum, leading to complete resolution without the need for surgical intervention. It also emphasizes the importance of careful monitoring, timely imaging, and individualized management strategies, particularly the recognition that hemodynamically stable patients may be managed conservatively.

## Data Availability

Not applicable. No datasets were generated or analyzed for this case report; all data supporting the findings are presented within the article.

## Research registration unique identifying number (UIN)

This case report doesn’t have a UIN.
